# Does exercise modulate ferroptosis-related cardiovascular injury? Mechanistic evidence and translational boundaries

**DOI:** 10.3389/fphys.2026.1899817

**Published:** 2026-08-24

**Authors:** Mengzhao Han, Haoran Huang, Yang Li, Lan Wang, Long Ji, Haibing Yin, Penglei Fan, Xinguo Yuan

**Affiliations:** 1 College of Education and Sports Sciences, Yangtze University, Jingzhou, China; 2 Institute of Physical Education, Shaanxi Normal University, Xi’an, China; 3 School of Sports Education, Tianjin University of Sport, Tianjin, China

**Keywords:** cardiomyopathy, cardiovascular injury, exercise training, exerkines, ferroptosis, GPX4, iron metabolism, lipid peroxidation

## Abstract

Ferroptosis connects disrupted iron handling, phospholipid peroxidation, and insufficient antioxidant defense with cardiovascular injury. Whether exercise can modify ferroptosis-related vulnerability in the heart and vasculature remains an important but incompletely resolved question. This mechanism-centered narrative review evaluates exercise-related evidence across cardiovascular injury contexts and distinguishes direct cardiovascular experiments from supportive marker studies, indirect mechanistic evidence, and human clinical contexts. PubMed was searched through June 2026, with supplementary retrieval from the Web of Science Core Collection and citation tracing. Among available exercise-specific studies, current evidence is most informative in preclinical models, particularly doxorubicin cardiotoxicity, where endurance exercise preconditioning has been linked to reduced iron accumulation, lipid-peroxide injury, mitochondrial damage, and myocardial dysfunction in studies incorporating ferroptosis-sensitive perturbation. Additional evidence from ischemia-reperfusion injury, diabetic cardiomyopathy, cardiac fibrosis, and experimental heart failure suggests that exercise may influence iron handling, the system Xc^−^–GSH–GPX4 axis, NRF2-related antioxidant regulation, lipid peroxidation, mitochondrial resilience, and inflammatory signaling. However, many studies remain marker-based, and evidence for resistance or combined training in tissue-level cardiovascular ferroptosis is limited. Human exercise studies provide translational context through improvements in vascular, metabolic, inflammatory, and oxidative-stress outcomes, but they do not yet establish ferroptosis modulation in cardiovascular tissue. Future studies require clearer exercise-dose reporting, serial sampling, tissue-level ferroptosis assays, pathway perturbation, clinically relevant models, and validated human biomarkers. Overall, exercise is best interpreted as a promising modifier of ferroptosis-related cardiovascular biology rather than an established ferroptosis-targeted therapy.

## 1 Introduction

Cardiovascular diseases (CVDs) encompass atherosclerotic vascular disease, myocardial infarction, heart failure, cardiomyopathies, hypertension-related injury, and other disorders that continue to impose substantial health and economic costs. Current prevention and treatment combine risk-factor control, pharmacotherapy, revascularization, device therapy, and rehabilitation, yet residual risk persists because structural damage, metabolic disturbance, inflammation, and cell death remain active across the disease course (Palaniappan et al., 2026). Exercise training is therefore used not as a substitute for evidence-based medical treatment but as a complementary intervention that can improve cardiorespiratory fitness, blood pressure, lipid handling, insulin sensitivity, endothelial function, and inflammatory status (Chen et al., 2022). These outcomes are clinically meaningful, but they do not by themselves identify the cellular pathways through which exercise modifies tissue vulnerability.

At the cellular level, the heart and vasculature are especially sensitive to changes in redox balance, mitochondrial function, inflammation, and iron and lipid metabolism because cardiomyocytes have high mitochondrial activity, endothelial cells are continuously exposed to hemodynamic and metabolic stress, and both cell types depend on tightly regulated metabolic homeostasis (Wang et al., 2022; Fang et al., 2023). Repeated exercise can enhance endogenous antioxidant capacity, improve mitochondrial quality control and substrate use, modulate inflammatory signaling, alter vascular shear stress, and regulate systemic mediators released from skeletal muscle and other organs (Chen et al., 2022; Zhang H. et al., 2024). Exercise may also influence transferrin receptor 1 (TfR1), ferritin, ferroportin (FPN), nuclear factor erythroid 2-related factor 2 (NRF2), solute carrier family 7 member 11 (SLC7A11), glutathione peroxidase 4 (GPX4), and acyl-CoA synthetase long-chain family member 4 (ACSL4), although the direction and magnitude of these responses vary according to the exercise protocol and cardiovascular injury model (Wang L. et al., 2024; Sabouri et al., 2025; Shen et al., 2025; Yang et al., 2025). Because these same homeostatic and stress-response pathways help determine whether cardiovascular cells adapt, recover, or die after injury, they provide a conceptual bridge from the established benefits of exercise to regulated cell death as a potential mechanism of cardioprotection.

Within this mechanistic context, regulated cell death has become an important framework for interpreting cardiovascular injury. Ferroptosis, first defined as an iron-dependent and non-apoptotic form of cell death, occurs when oxidizable phospholipids accumulate lethal peroxide damage in a setting of inadequate detoxification (Dixon et al., 2012). Its biochemical requirements distinguish it from apoptosis, necroptosis, and pyroptosis, although these processes may coexist in injured myocardium or vasculature. Three features are central: an expanded or poorly controlled labile iron pool, the presence of membrane phospholipids that can undergo chain-propagating peroxidation, and failure of antioxidant systems that normally reduce phospholipid hydroperoxides. Genetic loss of glutathione peroxidase 4 (GPX4) provided an early demonstration that impaired lipid-peroxide detoxification can initiate a distinct lethal program (Seiler et al., 2008; Friedmann Angeli et al., 2014; Yang et al., 2014). Recent cell-biological syntheses further emphasize that ferroptosis is a context-dependent constellation of iron, lipid, and redox processes rather than a single-marker phenotype (Dixon and Olzmann, 2024).

Ferroptosis is particularly relevant across cardiovascular injury contexts. Ischemia-reperfusion can rapidly alter redox balance and iron availability (Miyamoto et al., 2022); chronic heart failure can sustain mitochondrial dysfunction and inflammatory signaling; and atherosclerotic lesions combine lipid loading, disturbed flow, iron-rich microenvironments, and immune activation. These conditions can create changes compatible with ferroptotic vulnerability, but compatibility is not equivalent to proof. Increases in reactive oxygen species (ROS), malondialdehyde (MDA), 4-hydroxynonenal (4-HNE), ferritin, or total tissue iron may indicate oxidative injury or altered iron metabolism without establishing that ferroptotic cell death caused the phenotype. These mechanistic intersections have encouraged the proposal that exercise may protect the cardiovascular system partly by reducing ferroptotic susceptibility. The proposal is plausible, but the literature is heterogeneous and often relies on markers shared with general oxidative stress.

The distinction between direct and indirect evidence is especially important. A study that demonstrates rescue by ferrostatin-1 or liproxstatin-1, aggravation by erastin or RSL3, or a phenotype linked to genetic manipulation of GPX4, SLC7A11, or ACSL4 offers stronger causal support than a study that reports only lower MDA or higher GPX4 after training (Fang et al., 2023; Dixon and Olzmann, 2024). Likewise, findings from the brain, liver, or skeletal muscle may illuminate conserved pathways but cannot be presented as direct evidence of cardiovascular ferroptosis. Human exercise studies add translational context; yet, most measure fitness, endothelial function, circulating cytokines, or nonspecific oxidative markers rather than ferroptosis in cardiovascular tissue. Accordingly, contemporary reviews recommend convergent assays and causal perturbation before assigning a cardiovascular phenotype to ferroptosis (Zhang and Guo, 2023; Dixon and Olzmann, 2024).

This review first outlines core ferroptosis mechanisms relevant to cardiovascular injury, then considers their involvement across major cardiovascular contexts before evaluating exercise-related evidence and translational priorities. The synthesis distinguishes direct cardiovascular experiments from supportive marker studies, non-cardiovascular extrapolation, and the human clinical context so that mechanistic interpretation remains proportional to the evidence.

## 2 Review scope and evidence appraisal

### 2.1 Literature identification

This mechanism-centered narrative review used PubMed as the primary database, updated through 30 June 2026, with supplementary retrieval from the Web of Science Core Collection and backward and forward citation tracing. Search concepts combined ferroptosis and its principal molecular components with cardiovascular diagnoses and exercise terminology. Representative terms included ferroptosis, iron homeostasis, phospholipid peroxidation, GPX4, SLC7A11, ACSL4, FSP1, myocardial ischemia-reperfusion, heart failure, cardiomyopathy, atherosclerosis, endothelial injury, aerobic exercise, endurance training, resistance training, and high-intensity interval training (HIIT). Digital Object Identifiers and publication status were checked on publisher pages and Crossref records where available.

**Figure 1:**
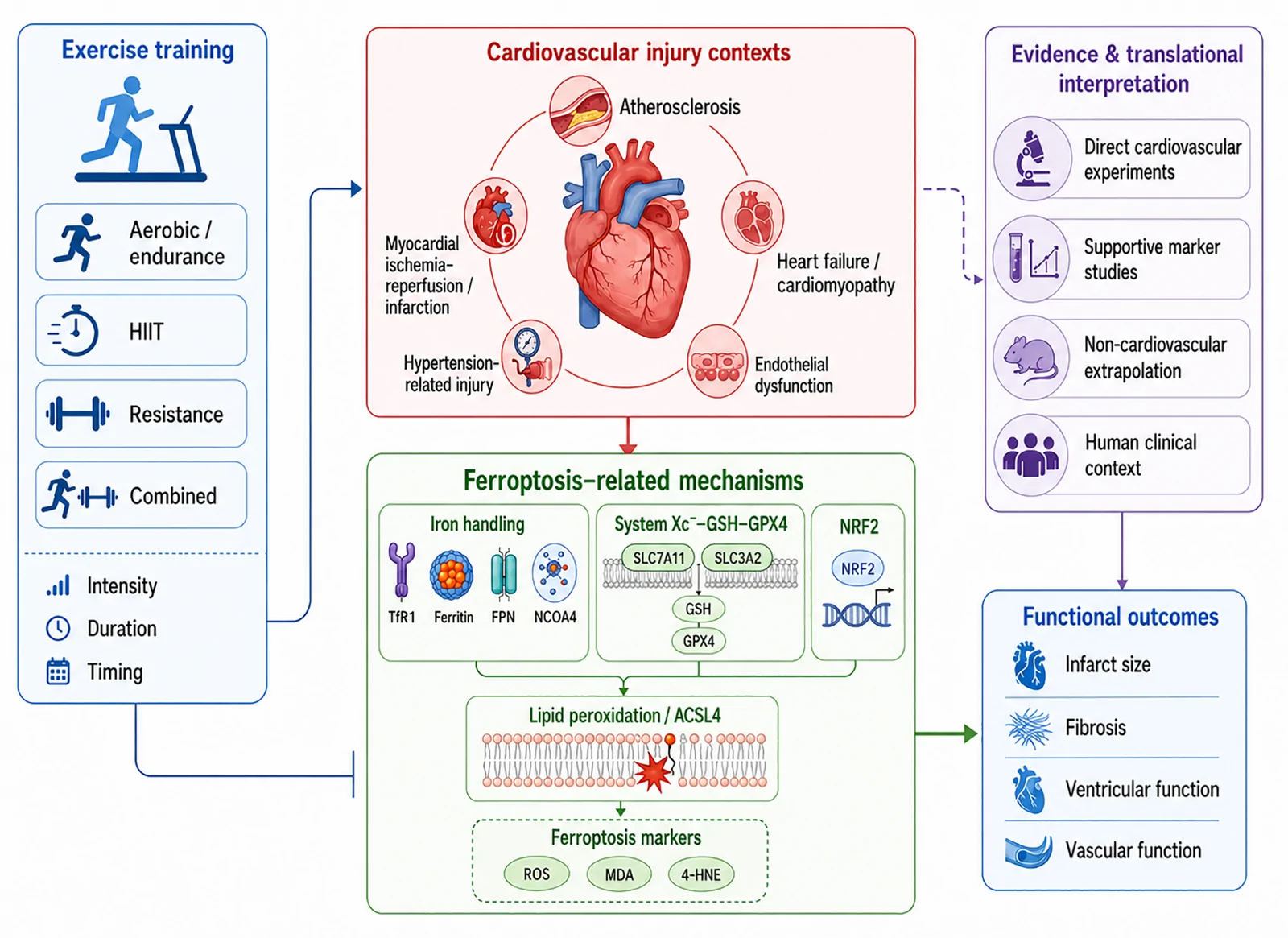
Conceptual framework linking exercise training, ferroptosis-related cardiovascular injury, and translational evidence. This conceptual framework illustrates how exercise exposure may influence ferroptosis-related cardiovascular biology across different disease contexts and levels of evidence. The blue module on the left summarizes exercise modalities, including aerobic/endurance training, high-intensity interval training, resistance exercise, and combined training, together with key dose characteristics such as intensity, duration, and timing. The red module in the upper center represents major cardiovascular injury contexts, including atherosclerosis, myocardial ischemia–reperfusion injury or myocardial infarction, heart failure or cardiomyopathy, hypertension-related injury, and endothelial dysfunction. The green module in the lower center summarizes the principal ferroptosis-related mechanisms potentially modified by exercise, including iron handling through TfR1, ferritin, FPN, and NCOA4; the system Xc^−^–GSH–GPX4 antioxidant axis; NRF2-related antioxidant regulation; ACSL4-associated phospholipid remodeling and lipid peroxidation; and supportive downstream markers such as ROS, MDA, and 4-HNE. The purple module on the upper right distinguishes four evidence categories used for interpretation: direct cardiovascular experiments, supportive marker studies, non-cardiovascular mechanistic extrapolation, and human clinical context. The blue module on the lower right summarizes functional cardiovascular outcomes, including infarct size, fibrosis, ventricular function, and vascular function. Solid arrows indicate proposed or experimentally supported directional relationships among exercise exposure, cardiovascular injury contexts, ferroptosis-related mechanisms, and functional outcomes. The blunt-ended blue line indicates a potential inhibitory or attenuating effect of exercise on ferroptosis-related injury mechanisms. The green arrow indicates that changes in ferroptosis-related mechanisms may contribute to cardiovascular functional outcomes. The dashed purple arrow indicates that evidence interpretation is applied to the mechanistic and disease-context relationships rather than representing a direct biological pathway. The vertical purple arrow indicates that evidence directness informs the interpretation of functional cardiovascular outcomes. Colors distinguish exercise exposure, cardiovascular injury contexts, ferroptosis-related mechanisms, evidence categories, and functional outcomes; they do not indicate evidence strength. ACSL4, acyl-CoA synthetase long-chain family member 4; 4-HNE, 4-hydroxynonenal; FPN, ferroportin; GPX4, glutathione peroxidase 4; GSH, glutathione; HIIT, high-intensity interval training; MDA, malondialdehyde; NCOA4, nuclear receptor coactivator 4; NRF2, nuclear factor erythroid 2-related factor 2; ROS, reactive oxygen species; SLC3A2, solute carrier family 3 member 2; SLC7A11, solute carrier family 7 member 11; TfR1, transferrin receptor 1; system Xc^−^, cystine/glutamate antiporter.

This mechanism-centered narrative review was designed to interpret the mechanistic directness and translational relevance of heterogeneous cardiovascular, exercise, and ferroptosis-related evidence. The literature was organized around three questions: (1) Which molecular events are sufficiently specific to support ferroptosis? (2) Which exercise experiments demonstrate, support, or indirectly suggest a change in ferroptotic susceptibility? (3) Which findings are sufficiently mature for cardiovascular translation? PubMed was used as the primary biomedical database, and Web of Science Core Collection and citation tracing were used to identify additional mechanistic and exercise-related studies. **Figure 1** provides an overview of the review framework, linking exercise exposure, cardiovascular injury contexts, ferroptosis-related mechanisms, functional outcomes, and evidence interpretation.

### 2.2 Selection principles and scope

Original animal, cellular, and human studies were prioritized when they examined ferroptosis in cardiovascular disease, paired an exercise intervention with ferroptosis-associated outcomes, or supplied mechanistic information necessary to interpret the exercise-cardiovascular connection. Relevant disease settings included myocardial ischemia-reperfusion injury, myocardial infarction, heart failure, diabetic and drug-induced cardiomyopathy, atherosclerosis, endothelial dysfunction, and hypertension-related injury. Aerobic or endurance training, exercise preconditioning, HIIT, resistance exercise, and combined programs were considered.

Non-cardiovascular exercise studies were retained when they tested mechanisms with plausible relevance to the heart or vasculature, such as exerkine signaling, iron transport, or GPX4-independent defense. These findings were interpreted as indirect mechanistic context rather than direct cardiovascular evidence. Human exercise studies were used primarily to inform translational relevance because available reports generally assess vascular function, systemic oxidative-stress markers, inflammation, metabolism, or candidate circulating mediators rather than tissue-level cardiovascular ferroptosis.

The review excludes unpublished observations and does not infer numerical precision from incomplete reports. Exercise dose, sex, sample size, tissue, timing, and ferroptosis assays were extracted when they were available in the article or accessible full text. When a field was absent or internally inconsistent, it was marked as not reported or described as a limitation. Marker-only studies were therefore interpreted as supportive rather than causal evidence.

### 2.3 Framework for judging ferroptosis evidence

Evidence directness was classified into four levels. Level A required a cardiovascular model, ferroptosis-specific rescue or induction, or manipulation of a core regulator, and convergent measurements of iron availability, phospholipid peroxidation, antioxidant failure, cell injury, or mitochondrial morphology. A single pharmacological inhibitor was not considered sufficient on its own because off-target effects remain possible. Level B required a cardiovascular model, measurements from at least two ferroptosis domains, and a cardiac or vascular outcome, but lacked a sufficiently specific rescue or genetic test.

Level C comprised limited marker panels, non-cardiovascular models used for mechanistic context, or findings that could reflect a broader antioxidant response. Level D was reserved for general redox evidence, such as changes in ROS, MDA, GSH, inflammatory mediators, or mitochondrial function without a ferroptosis-specific design. Accordingly, “demonstrated” is used only for Level A evidence; Level B is described as strongly supportive, whereas Levels C and D are presented as indirect or general redox findings. To make these distinctions explicit throughout the review, **Table 1** summarizes the evidence-directness framework and the wording used for each category.

**Table 1:** Evidence categories used in this narrative review.

Category	Interpretation	Minimum evidence	Permitted wording
A	Directly demonstrated	Cardiovascular model; ferroptosis-specific rescue/induction or genetic manipulation; convergent iron, phospholipid-peroxidation, antioxidant, and injury outcomes.	Demonstrated; causally supported.
B	Strongly supported	Cardiovascular model; at least two ferroptosis domains plus cardiac/vascular outcome; no sufficiently specific rescue or genetic validation.	Strongly supportive; consistent with reduced ferroptotic vulnerability.
C	Indirectly inferred	Limited marker panel, non-cardiovascular model, or mechanistic extrapolation.	Potential; indirect; mechanistically relevant.
D	General oxidative-stress evidence	ROS, MDA, GSH, inflammation, or mitochondrial measures without a ferroptosis-specific framework.	Reduced oxidative stress; not described as ferroptosis inhibition.

Categories describe the directness of ferroptosis evidence rather than overall methodological quality. The framework was used to guide wording throughout the review. Category A indicates direct demonstration with cardiovascular models, ferroptosis-specific rescue, induction, or genetic manipulation, and convergent injury-related outcomes. Category B indicates strongly supportive evidence but not definitive causal proof. Categories C and D indicate indirect mechanistic context or general oxidative-stress evidence and should not be described as direct ferroptosis inhibition.

GSH, glutathione; MDA, malondialdehyde; ROS, reactive oxygen species.

These categories describe mechanistic directness, not overall study quality. Timing, tissue compartment, exercise reporting, sample size, randomization, blinding, and outcome relevance were considered separately. Serum measurements were not treated as equivalent to myocardial or vascular tissue, and protein abundance was not treated as enzyme activity. No formal risk-of-bias score was applied because the evidence spans animal experiments, cell studies, and heterogeneous human designs.

## 3 Ferroptosis pathways relevant to cardiovascular injury

### 3.1 Iron homeostasis and the labile iron pool

Iron is essential for oxygen transport, mitochondrial respiration, enzymatic catalysis, and cellular signaling, but its redox activity requires strict control. Circulating ferric iron (Fe³^+^) is carried by transferrin and enters cells primarily through TfR1-mediated endocytosis. Endosomal reduction and divalent metal transporter 1 (DMT1) release ferrous iron (Fe²^+^) into the cytosol. Cytosolic iron may be incorporated into proteins, stored in ferritin, exported through FPN, or remain in a chelatable labile iron pool. When Fe²^+^ exceeds the buffering capacity of these systems, it can participate in Fenton chemistry and promote radical reactions that initiate or propagate lipid oxidation (Gao et al., 2015).

Ferritin limits iron toxicity by sequestering iron within a protein shell. Nuclear receptor coactivator 4 (NCOA4)-mediated ferritinophagy delivers ferritin to lysosomes and releases stored iron when cells require it. This process is physiologically useful, but excessive ferritinophagy can expand the labile iron pool and increase ferroptotic sensitivity (Mancias et al., 2014; Gao et al., 2016; Ajoolabady et al., 2021). Heme degradation can similarly release iron, and mitochondrial iron handling adds another compartment in which imbalance may become damaging. The direction of a ferritin change must therefore be interpreted in context: higher ferritin may reflect improved storage, an inflammatory response, or iron overload, whereas lower ferritin may reflect reduced iron burden or enhanced ferritinophagy.

In cardiovascular tissue, iron dysregulation can arise from ischemic injury, hemorrhage, heme turnover, chronic inflammation, metabolic disease, or cardiotoxic therapy. Myocardial ischemia-reperfusion has been associated with heme degradation, iron release, and ferroptosis-sensitive injury (Miyamoto et al., 2022). Population-level associations between serum ferritin and cardiovascular outcomes are clinically interesting but are not specific for ferroptosis because circulating ferritin is also affected by inflammation, liver function, infection, and systemic iron status (Klip et al., 2017). Direct inference requires tissue-level context and evidence that iron-dependent lipid damage contributes to cell death.

The method used to quantify iron also affects interpretation. Total iron assays do not distinguish protein-bound iron from the catalytically active fraction, and Prussian blue staining is less sensitive to rapidly exchangeable Fe²^+^ than fluorescent or chelation-based methods. TfR1, ferritin heavy chain 1, ferritin light chain, FPN, and NCOA4 describe different parts of iron traffic and may change in opposite directions as a compensatory response. A convincing ferroptosis study should therefore avoid reducing “iron homeostasis” to one protein. Ideally, it should pair a labile-iron measurement with at least one uptake, storage, release, or export measure and demonstrate that changing iron availability alters lipid-peroxide injury.

### 3.2 System Xc^−^–GSH–GPX4 axis

The cystine/glutamate antiporter system Xc^−^ is composed of the light-chain transporter SLC7A11 and the heavy-chain partner SLC3A2. It imports extracellular cystine in exchange for intracellular glutamate. Cystine is reduced to cysteine, which supports the synthesis of glutathione (GSH). GPX4 uses GSH-derived reducing equivalents to convert phospholipid hydroperoxides (PL-OOH) into the corresponding non-toxic phospholipid alcohols (PL-OH). Interruption at any point in this pathway can lower the threshold for ferroptosis: cystine deprivation or SLC7A11 inhibition limits GSH synthesis, GSH depletion weakens the reducing capacity, and GPX4 inactivation permits phospholipid hydroperoxides to accumulate (Dixon et al., 2014; Yang et al., 2014).

GPX4 is a central regulator, but its expression alone is not a binary readout of ferroptosis. Protein abundance does not necessarily report catalytic activity, substrate availability, membrane peroxide load, or the contribution of parallel defense pathways. Similarly, NRF2 can increase the expression of SLC7A11, ferritin, heme oxygenase-1, and other antioxidant genes, but NRF2 activation is a broad stress response rather than a ferroptosis-specific event (Dodson et al., 2019). In cardiovascular exercise models, aerobic treadmill training in diabetic cardiomyopathy and vitamin D-deficient fibrotic remodeling has been associated with activation of NRF2–GPX4-related signaling (Cui et al., 2026; Xie et al., 2026), whereas swimming training during doxorubicin exposure activated the miR-17-3p/KEAP1/NRF2 pathway (Gao et al., 2024). These modality- and model-specific findings support improved antioxidant defense, but they constitute strong ferroptosis evidence only when accompanied by appropriate iron, lipid-peroxide, and cell-death measurements or specific rescue experiments (Dixon and Olzmann, 2024; Wang L. et al., 2024; Sabouri et al., 2025; Shen et al., 2025; Yang et al., 2025).

The system Xc^−^–GSH–GPX4 axis also interacts with cellular metabolism. Cysteine availability, glutamate flux, NADPH regeneration, selenium status, and mitochondrial activity influence its function. In diseased myocardium, energetic failure and oxidative stress can impair multiple components simultaneously. Exercise may strengthen this axis through transcriptional adaptation, improved metabolic flexibility, or preservation of mitochondrial function, but the causal sequence should be tested rather than assumed (Shimada et al., 2016).

Redox measurements require similar discipline. Total GSH may remain stable even when the GSH/GSSG ratio falls, and GPX4 protein can be present while reducing equivalents are insufficient (Dixon et al., 2014; Dodson et al., 2019; Dixon and Olzmann, 2024). SLC7A11 upregulation may be adaptive, but it can also reflect cellular stress (Dixon et al., 2014; Dixon and Olzmann, 2024). NRF2 activation can increase heme oxygenase-1, which may be protective through antioxidant signaling yet may also release iron from heme under some conditions (Dodson et al., 2019; Fang et al., 2023). The same molecular change can therefore have different consequences depending on timing, magnitude, and the capacity of ferritin and FPN to handle newly available iron (Dodson et al., 2019; Fang et al., 2023; Dixon and Olzmann, 2024). Exercise studies should therefore measure the pathway as a functional system rather than as a list of isolated proteins (Dixon and Olzmann, 2024; Nakamura et al., 2024).

### 3.3 Phospholipid remodeling and lipid peroxidation

Ferroptotic injury depends on the oxidation of membrane phospholipids containing polyunsaturated fatty-acid chains. ACSL4 activates free polyunsaturated fatty acids to form acyl-CoA intermediates, and lysophosphatidylcholine acyltransferase 3 (LPCAT3) incorporates these chains into membrane phospholipids. This remodeling creates PUFA-containing phospholipids (PUFA-PLs) that are susceptible to oxidation. ACSL4 is therefore a determinant of ferroptotic sensitivity rather than a universal marker of cell death (Doll et al., 2017; Kagan et al., 2017).

PUFA-PL oxidation may proceed through enzymatic routes involving lipoxygenases (ALOX enzymes) or through non-enzymatic radical chain reactions initiated by iron-dependent chemistry. The resulting phospholipid hydroperoxides can fragment into reactive aldehydes, alter membrane curvature and permeability, damage proteins, and amplify oxidative reactions. MDA and 4-HNE are commonly measured downstream products, but they are generated in many oxidative conditions. Their presence supports lipid peroxidation, not necessarily ferroptosis. More specific approaches include oxidized phospholipid lipidomics, C11-BODIPY oxidation, assessment of GPX4 activity, and demonstration that a ferroptosis-directed intervention rescues cell viability or tissue function (Drummen et al., 2002; Wenzel et al., 2017; Wiernicki et al., 2020).

Cardiovascular diseases create abundant opportunities for lipid-peroxide formation. Atherosclerotic lesions contain oxidized lipids and iron-rich macrophage environments (Bai et al., 2020; Du et al., 2024; Hong et al., 2024; Tan et al., 2024; Jinson et al., 2025), while ischemic myocardium experiences abrupt changes in oxygen supply, redox state, and iron availability (Park et al., 2019; Miyamoto et al., 2022; Cai et al., 2023). Doxorubicin disrupts mitochondrial and iron metabolism and can promote ferroptosis-related cardiotoxicity (Tadokoro et al., 2020). These settings may share a common lipid-peroxide pathway, but their initiating events and dominant cell types differ (Fang et al., 2023; Zhang and Guo, 2023; Liu et al., 2024). Endothelial cells, cardiomyocytes, fibroblasts, vascular smooth muscle cells, and macrophages should therefore not be treated as interchangeable (Fang et al., 2023; Zhang and Guo, 2023; Liu et al., 2024; Jinson et al., 2025).

Lipid composition is another source of heterogeneity. The abundance and location of arachidonic- or adrenic-acid-containing phospholipids influence susceptibility, as do membrane remodeling, dietary fatty acids, and cellular differentiation. ACSL4 suppression can lower ferroptotic sensitivity in many settings, but ACSL4 also participates in normal lipid metabolism. Likewise, a reduction in circulating triglycerides after training does not establish a change in oxidizable myocardial phospholipids. Studies that connect exercise to ferroptosis should distinguish systemic lipid profiles from the specific membrane species that undergo peroxidation (Sun et al., 2021).

### 3.4 GPX4-independent defense pathways

Cells possess additional mechanisms that restrain phospholipid peroxidation. Ferroptosis suppressor protein 1 (FSP1) uses NAD(P)H to maintain coenzyme Q in its reduced form, CoQH_2_, at cellular membranes. Reduced coenzyme Q can interrupt lipid-radical propagation and operate in parallel with GPX4 (Bersuker et al., 2019). The GTP cyclohydrolase 1-tetrahydrobiopterin (GCH1–BH_4_) pathway also supports membrane redox balance through lipid remodeling (Kraft et al., 2020), and mitochondrial dihydroorotate dehydrogenase (DHODH) contributes to CoQ-related ferroptosis defense within mitochondria (Wang C. et al., 2024). These pathways help explain why cells with similar GPX4 expression may differ in ferroptosis sensitivity (Bersuker et al., 2019; Kraft et al., 2020; Wang C. et al., 2024).

Among the cardiovascular exercise studies summarized in **Table 2**, available reports have primarily assessed iron handling, NRF2-related regulation and system Xc^−^–GSH–GPX4 signaling, ACSL4, or general redox markers rather than FSP1–CoQ, GCH1–BH_4_, or DHODH (Seiler et al., 2008; Friedmann Angeli et al., 2014; Yang et al., 2014; Gao et al., 2024; Liu H. et al., 2025; Sabouri et al., 2025; Cui et al., 2026; Xie et al., 2026). Claims that a specific exercise modality activates these GPX4-independent defenses should therefore be limited to studies that directly assess the relevant components. At present, these pathways are better presented as plausible extensions of exercise-responsive redox adaptation than as established exercise mechanisms.

**Table 2:** Cardiovascular exercise studies with ferroptosis-related outcomes and evidence appraisal.

Study	Model/species/sex/sample size	Exercise exposure	Tissue/cell system	Ferroptosis-related assays and cardiovascular outcomes	Evidence appraisal
(Seiler et al., 2008)	Doxorubicin-induced cardiotoxicity; male C57BL/6J mice; 12 mice/group in the main *in-vivo* comparison, with smaller subsets used for selected assays	Treadmill endurance preconditioning for 4 weeks, continued during 3 weeks of doxorubicin exposure; zero incline; running speed NR	Left-ventricular myocardium and primary adult mouse cardiomyocytes	Ferrostatin-1, erastin, AMPKα2 knockdown/inhibition, and autophagy-modulating interventions; Fe²^+^, MDA, 4-HNE, GPX4, PTGS2, ROS, mitochondrial complex-I activity, and ultrastructure; LVEF, LVFS, and myocardial injury	Category A. The most comprehensive causal design, combining ferroptosis induction and inhibition with pathway manipulation and cardiac outcomes. Drug-induced injury and the preconditioning design limit generalization to established CVD.
(Yang et al., 2014)	Myocardial ischemia–reperfusion; male C57BL/6J, IGFBP2-knockout, and cardiomyocyte-specific IGF-1R-knockout mice, 6–8 weeks old; 80 mice in eight groups, 10/group	Treadmill exercise at 50% VO_2_max, 55 min/day, 5 days/week. The Methods specify 4 weeks, whereas several figure legends describe 8 weeks	Mouse myocardium and plasma; primary neonatal mouse cardiomyocytes subjected to hypoxia/reoxygenation	IGFBP2 knockout, cardiomyocyte-specific IGF-1R knockout, and SIRT1–TXNIP/TRX manipulation; Fe²^+^, MDA, LPO, 4-HNE, 4-HHE, ROS, GSH, GPX4, PGC-1α, and cell viability; echocardiography, exercise capacity, infarct size, and myocardial fibrosis	Category B. Strongly supportive evidence indicates that IGFBP2–IGF-1R signaling is required for the exercise response. The inconsistent intervention duration and absence of ferroptosis-specific rescue or core-effector manipulation prevent definitive causal attribution.
(Friedmann Angeli et al., 2014)	High-fat diet/streptozotocin-induced diabetic cardiomyopathy; male and female mice, including cardiomyocyte-specific NAT10-knockout mice; overall group size NR, with several outcome analyses reporting n=5/group; explanted human hearts	Forced treadmill training for 8 weeks; detailed workload was referenced to an earlier protocol and was not fully reproduced in the brief report	Mouse myocardium and explanted human myocardial tissue	Cardiomyocyte-specific NAT10 deletion and ac^4^C-RIP-seq; NAT10-dependent RNA N^4^-acetylcytidine modification; FTH1, SLC7A11, GPX4, and PTGS2; systolic and diastolic cardiac function; NAT10/ac^4^C and ferroptosis-defense proteins in human myocardial tissue	Category B. Cardiomyocyte NAT10 was necessary for the exercise-associated cardiac response. The brief-report format, incomplete exercise-dose reporting, outcome-specific rather than total sample sizes, and the absence of a core ferroptosis rescue limit causal attribution.
(Yang et al., 2022)	Diabetic cardiomyopathy; 30 male Sprague–Dawley rats randomized to three groups, 10/group; high-glucose/palmitate-treated H9c2 cells	One-week treadmill adaptation followed by 8 weeks at 15.2 m/min, slope setting 3, approximately 58% VO_2_max, 60 min/day, 5 days/week	Left-ventricular myocardium and high-glucose/palmitate-treated H9c2 cardiomyoblasts	P4HA1 and HIF-1α overexpression or knockdown; NRF2/GPX4 signaling, lipid-peroxidation indices, oxidative-stress markers, mitochondrial morphology, myocardial histopathology, fibrosis, and cardiac function	Category B. Molecular perturbation supports an exercise-responsive P4HA1–HIF-1α–NRF2/GPX4 pathway. The male-only design and the proposed relationship between P4HA1 and HIF-1α stability require independent confirmation and direct enzyme-activity measurements.
(Kuppe et al., 2022)	Vitamin D deficiency-associated cardiac fibrosis; mice with and without cardiac-specific VDR knockdown; sex and group size NR	Treadmill exercise for 12 weeks following dietary induction of vitamin D deficiency; detailed speed, incline, and session frequency NR	Myocardium from vitamin D-deficient and cardiac-specific VDR-knockdown mice	Cardiac VDR knockdown; VDR–NRF2–GPX4 signaling, ferroptosis-associated markers, mitochondrial abnormalities, collagen deposition, myocardial fibrosis, and cardiac remodeling	Category B. Cardiac VDR knockdown supports the pathway necessity for the exercise-associated response. The lack of core ferroptosis rescue and incomplete exercise-dose and sample reporting restrict mechanistic certainty.
(Zeng et al., 2023)	Doxorubicin-induced cardiotoxicity; male C57BL/6J mice: control n=6, doxorubicin n=10, exercise n=6, and doxorubicin plus exercise n=10; H9c2 cells	Aerobic exercise for 8 weeks during and after doxorubicin administration; intensity and weekly frequency incompletely reported	Mouse myocardium and doxorubicin-treated H9c2 cardiomyoblasts	Fe²^+^, ROS, total lipid peroxides, MDA, 4-HNE, FTH1, TfR1, SLC7A11, and ACSL4; myocardial fibrosis, cTnT, LVEF, LVFS, and survival; ferrostatin-1 tested in H9c2 cells	Category B. Multi-domain myocardial and functional evidence is consistent with reduced ferroptotic susceptibility. Ferrostatin-1 was tested only *in vitro*, and the exercise effect was not reversed through a ferroptosis-specific intervention *in vivo*.
(Li F-J. et al., 2024)	Doxorubicin-induced cardiomyopathy in mice; sex and group size NR	Swimming training; duration, frequency, session duration, and workload incompletely reported	Mouse myocardium; *in-vivo* miR-17-3p inhibition	miR-17-3p inhibition and KEAP1/NRF2 signaling; myocardial Fe²^+^, MDA, GSH, GPX4, and SLC7A11; cardiac function and myocardial injury	Category B. Inhibition of miR-17-3p weakened the cardiac benefit of swimming and reversed several ferroptosis-associated changes. Incomplete exercise and sample reporting and the absence of a ferroptosis-specific rescue remain important limitations.
(Dixon and Olzmann, 2024)	Isoprenaline-induced heart failure; 24 male Wistar rats, three groups with 8 rats/group	HIIT for 8 weeks: five 4-min intervals at 85%–90% VO_2_max, separated by 2-min active recovery at 50%–60% VO_2_max; warm-up and cool-down included	Left-ventricular myocardium; circulating samples used for myocardial-injury biomarkers	Myocardial iron deposition, TfR1, FPN1, ferritin, MDA, SLC7A11, GPX4, NRF2, and PERK/ATF4-associated ER-stress proteins; EF, FS, myocardial fibrosis, cTnI, and LDH	Category B. The study combined multiple ferroptosis domains with structural and functional cardiac outcomes. The absence of pharmacological or genetic rescue, the male-only sample, and the lack of an energy-matched continuous-training group limit causal and modality-specific conclusions.

Categories describe the directness of ferroptosis evidence rather than overall methodological quality. Exercise dose, sample size, sex, tissue or cell system, cardiovascular outcomes, and ferroptosis-specific validation were considered when available. Studies classified as Category B were interpreted as strongly supportive rather than definitive evidence of ferroptotic causality. Incomplete exercise-dose reporting, missing sex or sample-size information, and lack of ferroptosis-specific rescue or genetic validation were treated as limitations.

ac^4^C, N^4^-acetylcytidine; ACSL4, acyl-CoA synthetase long-chain family member 4; AMPKα2, AMP-activated protein kinase alpha 2; cTnI, cardiac troponin I; cTnT, cardiac troponin T; EF, ejection fraction; ER, endoplasmic reticulum; FPN1, ferroportin 1; FS, fractional shortening; FTH1, ferritin heavy chain 1; GSH, glutathione; H/R, hypoxia/reoxygenation; HIIT, high-intensity interval training; IGF-1R, insulin-like growth factor 1 receptor; IGFBP2, insulin-like growth factor-binding protein 2; LDH, lactate dehydrogenase; LPO, lipid peroxidation; LVEF, left ventricular ejection fraction; LVFS, left ventricular fractional shortening; MDA, malondialdehyde; NAT10, N-acetyltransferase 10; NR, not reported; P4HA1, prolyl 4-hydroxylase subunit alpha 1; ROS, reactive oxygen species; TfR1, transferrin receptor 1; VDR, vitamin D receptor; 4-HHE, 4-hydroxyhexenal; 4-HNE, 4-hydroxynonenal.

### 3.5 Mitochondrial dysfunction, inflammation, and cell-death crosstalk

Mitochondria can influence ferroptosis through energy metabolism, iron handling, ROS production, and membrane lipid composition. Their contribution varies with the initiating stimulus. In some contexts, mitochondrial metabolism is required for ferroptosis; in others, plasma-membrane and endoplasmic-reticulum lipid oxidation dominate (Gao et al., 2019). In the heart, mitochondrial dysfunction is especially relevant because cardiomyocytes rely heavily on oxidative phosphorylation. Loss of mitochondrial membrane potential, impaired electron transport, and altered tricarboxylic-acid-cycle flux can increase oxidative pressure and weaken cellular redox recovery, thereby increasing susceptibility to ferroptosis-related injury (Gao et al., 2019; Ajoolabady et al., 2021).

Ferroptosis also intersects with inflammatory signaling. Lipid-peroxidation products and damage-associated molecular patterns may activate innate immune pathways, including Toll-like receptor 4 and the NLRP3 inflammasome, whereas inflammatory mediators can modify iron uptake, ferritin turnover, and ROS-generating enzymes (Cai et al., 2023; Zhang and Guo, 2023; Liu H. et al., 2025). These reciprocal interactions may establish a feed-forward loop that amplifies tissue injury in chronic cardiovascular disease. Nevertheless, inflammatory changes should be interpreted as complementary evidence rather than as ferroptosis-specific evidence unless iron-dependent phospholipid peroxidation and ferroptosis-sensitive perturbation are also demonstrated (Zhang and Guo, 2023; Shen et al., 2025; Yang et al., 2025).

**Figure 2:**
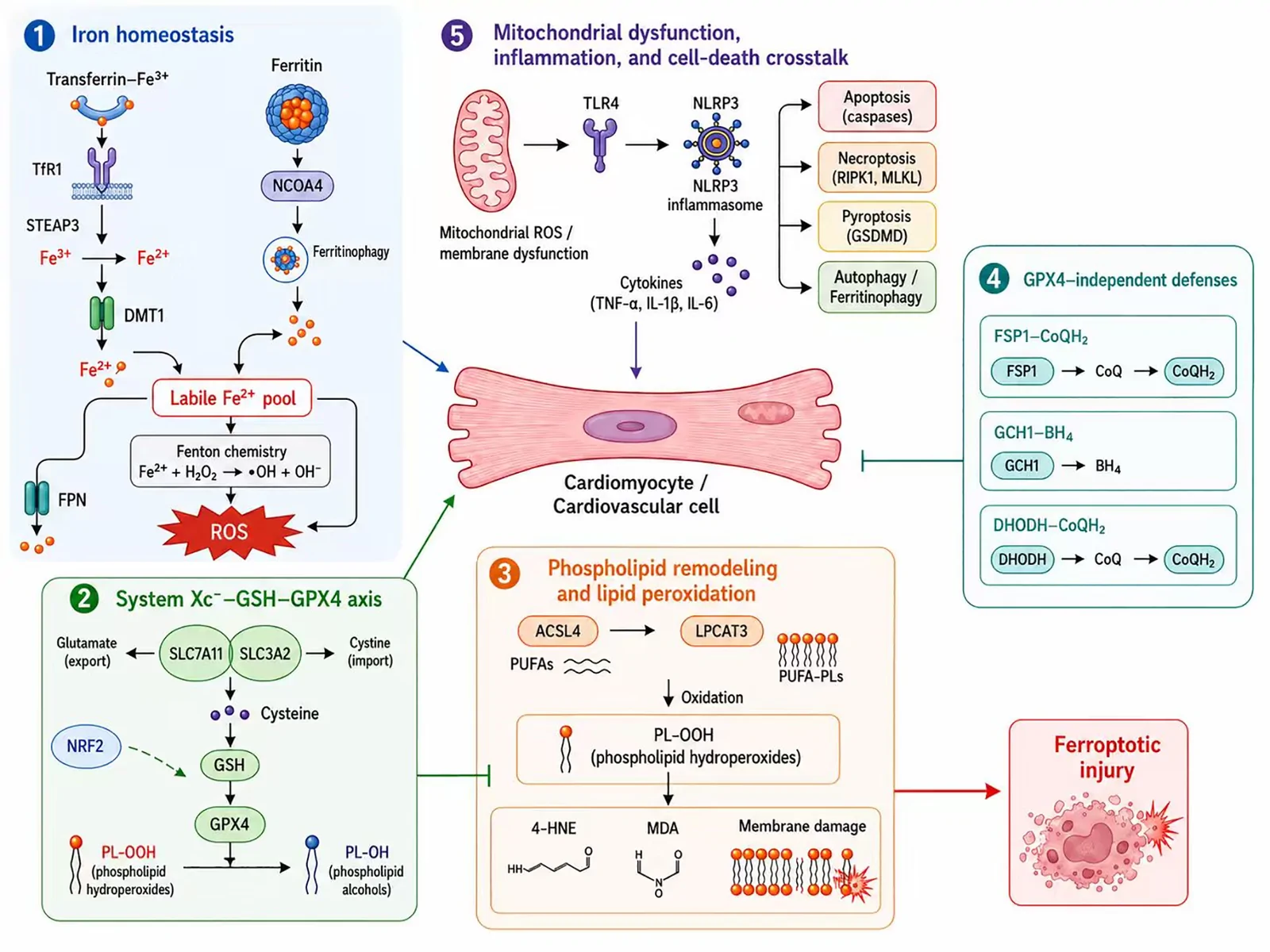
Molecular basis of ferroptosis in cardiovascular tissue. This schematic summarizes five interrelated mechanisms that regulate ferroptosis-related vulnerability and injury in cardiomyocytes and other cardiovascular cells. (1) Iron homeostasis. Transferrin-bound Fe³^+^ is internalized through TfR1-mediated endocytosis, reduced by STEAP3, and transported into the cytosol through DMT1 as Fe²^+^. Cytosolic iron may enter the labile Fe²^+^ pool, be stored in ferritin, released through NCOA4-mediated ferritinophagy, or exported through FPN. Excess labile Fe²^+^ promotes Fenton chemistry and ROS production, thereby increasing oxidative pressure and ferroptosis-related susceptibility. (2) System Xc^−^–GSH–GPX4 antioxidant axis. System Xc^−^, composed of SLC7A11 and SLC3A2, imports cystine in exchange for glutamate. Cystine is reduced to cysteine and supports GSH synthesis. GPX4 uses GSH-derived reducing equivalents to convert phospholipid hydroperoxides (PL-OOH) into non-toxic phospholipid alcohols (PL-OH). NRF2 supports this antioxidant system through transcriptional regulation of redox- and iron-related genes. Impairment of system Xc^−^, GSH availability, or GPX4 activity permits phospholipid hydroperoxides to accumulate. (3) Phospholipid remodeling and lipid peroxidation. ACSL4 activates polyunsaturated fatty acids, and LPCAT3 incorporates them into membrane phospholipids, forming oxidation-sensitive PUFA-containing phospholipids. Their oxidation generates PL-OOH, reactive aldehydes such as 4-HNE and MDA, and membrane damage, thereby promoting ferroptotic injury. (4) GPX4-independent defense pathways. FSP1 maintains CoQ in its reduced antioxidant form, CoQH_2_, at cellular membranes. The GCH1–BH_4_ pathway supports membrane antioxidant capacity, while mitochondrial DHODH reduces CoQ to CoQH_2_ within mitochondria. These parallel defenses restrain phospholipid-peroxide propagation independently of GPX4. (5) Mitochondrial dysfunction, inflammation, and cell-death crosstalk. Mitochondrial ROS production and membrane dysfunction can amplify oxidative injury. TLR4- and NLRP3-related inflammatory signaling promotes cytokine release, including TNF-α, IL-1β, and IL-6, and may interact with iron handling and lipid peroxidation. Ferroptosis may also coexist or interact with apoptosis, necroptosis, pyroptosis, autophagy, and ferritinophagy. Black arrows indicate molecular transport, biochemical conversion, or directional signaling within individual pathways. The blue arrow indicates the contribution of iron-dependent oxidative stress to cardiovascular cell injury, whereas the purple arrow indicates inflammatory signaling toward the cardiomyocyte or cardiovascular cell. Green arrows indicate protective or regulatory effects associated with the system Xc^−^–GSH–GPX4 axis. Green and teal blunt-ended lines indicate inhibitory effects of GPX4-dependent and GPX4-independent antioxidant defenses on lipid-peroxide accumulation or ferroptosis-related cellular injury. The red arrow indicates progression from phospholipid peroxidation and membrane damage toward ferroptotic injury. The numbered, color-coded sections distinguish iron homeostasis, the system Xc^−^–GSH–GPX4 axis, phospholipid remodeling and lipid peroxidation, GPX4-independent defenses, and mitochondrial, inflammatory, and cell-death crosstalk. Colors identify mechanistic domains and do not indicate evidence strength. ACSL4, acyl-CoA synthetase long-chain family member 4; BH_4_, tetrahydrobiopterin; CoQ, coenzyme Q; CoQH_2_, reduced coenzyme Q; DHODH, dihydroorotate dehydrogenase; DMT1, divalent metal transporter 1; Fe²^+^, ferrous iron; Fe³^+^, ferric iron; FPN, ferroportin; FSP1, ferroptosis suppressor protein 1; GCH1, GTP cyclohydrolase 1; GPX4, glutathione peroxidase 4; GSH, glutathione; 4-HNE, 4-hydroxynonenal; IL-1β, interleukin-1 beta; IL-6, interleukin-6; LPCAT3, lysophosphatidylcholine acyltransferase 3; MDA, malondialdehyde; NCOA4, nuclear receptor coactivator 4; NLRP3, NLR family pyrin domain containing 3; NRF2, nuclear factor erythroid 2-related factor 2; PL-OH, phospholipid alcohol; PL-OOH, phospholipid hydroperoxide; PUFA-PLs, polyunsaturated fatty acid-containing phospholipids; ROS, reactive oxygen species; SLC3A2, solute carrier family 3 member 2; SLC7A11, solute carrier family 7 member 11; STEAP3, six-transmembrane epithelial antigen of prostate 3; TfR1, transferrin receptor 1; TLR4, Toll-like receptor 4; TNF-α, tumor necrosis factor alpha; system Xc^−^, cystine/glutamate antiporter.

Apoptosis, necroptosis, pyroptosis, autophagy, and ferroptosis may occur in parallel or sequentially (Zhang and Guo, 2023). Autophagy can protect cells by removing damaged organelles, whereas ferritinophagy can increase iron release (Mancias et al., 2014; Gao et al., 2016; Ajoolabady et al., 2021). Exercise-induced autophagy may therefore be beneficial or harmful depending on timing, intensity, and substrate. The major ferroptosis-related pathways discussed in this section are summarized in **Figure 2**.

## 4 Ferroptosis across cardiovascular disease contexts

### 4.1 Myocardial ischemia-reperfusion injury

Myocardial ischemia-reperfusion injury combines oxygen deprivation, metabolic arrest, and abrupt reoxygenation. Reperfusion is essential for salvaging ischemic tissue, but it can generate ROS, disturb calcium and iron handling, and accelerate membrane oxidation. Experimental work has linked heme degradation and iron release to ferroptosis-sensitive myocardial injury, providing a biologically coherent route from reperfusion to lipid-peroxide accumulation (Feng et al., 2019; Park et al., 2019; Miyamoto et al., 2022; Sun et al., 2022; Xie et al., 2022; Zhu et al., 2024; Wang et al., 2025). Other studies report lower GPX4 or SLC7A11, higher ACSL4, increased Fe²^+^, and accumulation of 4-HNE or MDA. Recent syntheses emphasize that ferroptosis coexists and cross-regulates with apoptosis, necroptosis, pyroptosis, and autophagy during myocardial reperfusion injury (Fang et al., 2023; Zhang and Guo, 2023; Du et al., 2025).

Ferroptosis-directed interventions have reduced myocardial injury in experimental models. In diabetic ischemia-reperfusion injury, DNA methyltransferase 1 and NCOA4-mediated ferritinophagy were linked to iron release and tissue damage (Li et al., 2021). In separate cardiac models, ferrostatin-1 and iron chelation limited doxorubicin- and ischemia-reperfusion-associated injury, supporting a functional role for iron-dependent lipid damage rather than a marker-only association (Fang et al., 2019; Feng et al., 2019; Tadokoro et al., 2020). The magnitude and timing of this contribution nevertheless vary with the model and the coexistence of apoptosis, necrosis, and inflammation.

Most preclinical myocardial ischemia-reperfusion studies use young rodents or isolated-cell systems, controlled ischemic periods, and relatively short observation windows (Feng et al., 2019; Park et al., 2019; Li et al., 2021; Miyamoto et al., 2022; Sun et al., 2022; Xie et al., 2022; Yang et al., 2022; Cai et al., 2023; Chen et al., 2023; Mohammadkhani et al., 2023; Zhu et al., 2024; Du et al., 2025; Wang et al., 2025). Human myocardial infarction, by contrast, occurs in clinically heterogeneous patients, and spatial multi-omic studies of human infarct tissue demonstrate complex, compartment-specific immune and tissue remodeling that is not reproduced by simplified rodent protocols (Kuppe et al., 2022; Wünnemann et al., 2025). A ferroptosis-associated marker measured after injury may therefore reflect damage already sustained rather than a tractable therapeutic window (Dixon and Olzmann, 2024; Du et al., 2025). Exercise-preconditioning experiments are mechanistically informative (Mohammadkhani et al., 2023; Yang et al., 2025), but by design they model protection acquired before ischemia and do not replicate the clinical presentation of an untrained patient with an acute coronary syndrome.

The temporal profile of ferroptosis after reperfusion also remains uncertain. Early iron release and lipid-radical formation may occur within minutes to hours, whereas inflammatory amplification and remodeling continue for days. A single terminal measurement cannot show whether exercise delayed the process, reduced its peak, accelerated recovery, or simply altered baseline protein expression. Serial sampling and cell-type-resolved methods are needed. Spatial transcriptomics, targeted lipid imaging, and single-cell approaches could identify whether cardiomyocytes, endothelial cells, fibroblasts, or infiltrating immune cells are the principal exercise-responsive compartment. This temporal and cellular heterogeneity is increasingly recognized as a major barrier to mechanism-specific intervention (Dixon and Olzmann, 2024; Du et al., 2025).

Recent exercise work has extended the ischemia-reperfusion literature beyond general antioxidant adaptation. In mice, an exercise-responsive circulating IGFBP2 signal was linked to smaller infarcts, less fibrosis, and a multi-domain ferroptosis-associated profile; loss of IGFBP2 or cardiomyocyte IGF-1 receptor weakened the benefit (Yang et al., 2025). Because this pathway also regulates broader redox and metabolic processes, the experiment is best viewed as mechanistically informative and strongly supportive rather than definitive evidence of ferroptotic causality.

### 4.2 Myocardial infarction

Myocardial infarction includes ischemic necrosis and, when blood flow is restored, a component of reperfusion injury. Ferroptosis-related processes may be most active in the peri-infarct region, where cells experience severe oxidative and metabolic stress but have not yet undergone irreversible structural destruction. Iron accumulation, reduced GSH-GPX4 capacity, ACSL4-dependent phospholipid remodeling, and mitochondrial dysfunction can lower the survival threshold of these cells (Park et al., 2019).

The evidence base includes pharmacological and genetic studies, but terminology is not always consistent. A reduction in infarct size accompanied by lower MDA and higher GPX4 should be described as compatible with reduced ferroptotic vulnerability unless specific rescue or induction is shown (Cai et al., 2023; Chen et al., 2023). This distinction matters because many cardioprotective interventions also reduce inflammation, calcium overload, apoptosis, and mitochondrial permeability transition (Cai et al., 2023). A causal claim should demonstrate that manipulating ferroptosis changes the cardiovascular phenotype independently or as a necessary component of the broader response (Cai et al., 2023; Chen et al., 2023).

Exercise may influence the myocardial infarct response through enhanced antioxidant capacity, mitochondrial conditioning, autonomic regulation, vascular adaptation, and inflammatory resolution (Chen et al., 2022; Mohammadkhani et al., 2023; Zhang H. et al., 2024). These mechanisms may intersect with ferroptosis-related pathways but cannot be reduced to ferroptosis alone. Studies of exercise after myocardial infarction should therefore report the timing of training relative to injury, because preconditioning, early rehabilitation, and chronic post-infarction training address distinct biological questions and carry different clinical and safety implications (Yang et al., 2014; Mohammadkhani et al., 2023; Zhang H. et al., 2024).

### 4.3 Heart failure, cardiomyopathy, and fibrotic remodeling

Heart failure is a syndrome rather than a single disease mechanism. Pressure overload, ischemic injury, cardiotoxic drugs, genetic cardiomyopathy, metabolic disease, and inflammation can all produce ventricular dysfunction. Persistent mitochondrial stress, altered substrate use, iron imbalance, and inadequate antioxidant defense create conditions in which ferroptosis-related pathways may remain active. Experimental heart-failure studies report changes in NRF2, SLC7A11, GPX4, ACSL4, TfR1, ferritin, FPN, and lipid-peroxidation products (Wang C. et al., 2024). Recent reviews further characterize heart failure and cardiomyopathy as heterogeneous settings in which iron, lipid, mitochondrial, and inflammatory pathways contribute with different weights (Fang et al., 2023; Zhang and Guo, 2023; Liu et al., 2024).

NOX4-dependent oxidative signaling has been linked to cell death and ferroptosis-associated changes in heart failure models (Chen et al., 2019). Manipulation of the miR-432-5p-Keap1-NRF2-SLC7A11 axis has also been reported to alter hypoxia-reoxygenation injury in cardiomyocytes (Geng et al., 2023). Pharmacological studies, including work on dapagliflozin, suggest that NRF2–HO-1–GPX4 signaling may accompany improved cardiac function (Zhang J. et al., 2025). These findings identify tractable pathways, but several are Level B or C because they rely on marker panels without a ferroptosis-specific rescue.

Doxorubicin-induced cardiomyopathy has produced some of the most informative exercise-ferroptosis experiments. Doxorubicin perturbs iron and mitochondrial metabolism and increases lipid-peroxide stress (Camilli et al., 2024). Because exercise can be introduced before and during exposure, investigators can test whether training modifies susceptibility rather than only responding to established failure. Even in this model, clinical translation must account for cancer type, treatment schedule, fatigue, anemia, and individual exercise tolerance.

Iron biology in heart failure is clinically complex because patients may have systemic iron deficiency while specific tissues or subcellular compartments remain oxidatively stressed (Graham et al., 2024; Packer et al., 2024). Treating ferritin or transferrin saturation as a surrogate for myocardial labile iron can therefore be misleading. Iron supplementation can improve symptoms in selected patients with heart failure and iron deficiency, while experimental iron overload can aggravate injury. A ferroptosis-oriented interpretation must preserve this distinction and should not imply that reducing systemic iron is generally beneficial. Exercise studies should report hematologic and systemic iron status when iron-related mechanisms are central to the hypothesis (Fefelova et al., 2023).

Other cardiomyopathies show related but distinct biology. In septic cardiomyopathy, inflammatory signaling and mitochondrial dysfunction interact with ferroptosis-associated pathways; resveratrol-mediated SIRT1-NRF2 activation has been linked to reduced injury (Zeng et al., 2023). Loss of FUNDC1-mediated mitochondrial quality control can intensify ACSL4-dependent ferroptosis in septic cardiomyopathy (Li F-J. et al., 2024). These studies support mechanistic crosstalk but do not demonstrate that a single ferroptosis pathway explains all cardiomyopathy phenotypes (Anker et al., 2009; Ponikowski et al., 2020).

Diabetic cardiomyopathy is an emerging exercise-ferroptosis evidence cluster. Treadmill studies have implicated NAT10-dependent RNA acetylation and a proposed P4HA1–HIF-1α–NRF2/GPX4 pathway, with molecular perturbations accompanying improvements in cardiac function (Zhang X. et al., 2024; Shen et al., 2025; Xie et al., 2026). A 2026 study in vitamin D-deficient mice further linked exercise to cardiac vitamin D receptor-NRF2-GPX4 signaling and reduced fibrotic remodeling; cardiac VDR knockdown weakened the response (Cui et al., 2026). These pathways are considered together in Section 5.3 because each is pleiotropic and requires additional testing against a core ferroptosis effector.

### 4.4 Atherosclerosis

Atherosclerosis develops through lipid retention, endothelial dysfunction, immune-cell recruitment, foam-cell formation, smooth-muscle remodeling, and plaque destabilization. Ferroptosis may contribute at several stages because oxidizable lipids, disturbed iron handling, inflammatory ROS, and compromised antioxidant defenses coexist in lesions (Du et al., 2024; Jinson et al., 2025). The effect is cell specific: endothelial ferroptosis may impair barrier function, macrophage ferroptosis may alter inflammation and necrotic-core development, and smooth-muscle-cell loss may weaken plaque structure. Recent cardiovascular syntheses similarly stress that the consequences of ferroptosis vary among endothelial cells, macrophages, and vascular smooth muscle cells (Fang et al., 2023; Zhang and Guo, 2023; Liu et al., 2024).

In experimental atherosclerosis, inhibition of ferroptosis reduced lipid peroxidation and endothelial dysfunction (Bai et al., 2020; Du et al., 2024). Berberine was reported to inhibit ACSL4-associated endothelial ferroptosis and reduce lesion burden (Hong et al., 2024), while atorvastatin altered ACSL4 and GPX4-related signaling in endothelial injury (Tan et al., 2024). These studies support a relationship between ferroptosis-associated pathways and lesion biology, but drug pleiotropy and the use of multiple cell types complicate causal attribution.

Human evidence is more limited. Circulating long non-coding RNA NORAD has been associated with endothelial-cell survival and GPX4-related signaling in coronary artery disease (He et al., 2024). Serum ferritin and lipid-peroxidation products may correlate with cardiovascular risk, but they lack sufficient specificity for diagnosing ferroptosis. Plaque imaging, vascular function, and conventional biomarkers remain clinically established, whereas no ferroptosis-specific diagnostic panel has yet been clinically validated for cardiovascular disease.

Cell type is likely to determine how ferroptosis shapes atherosclerosis. Endothelial loss can increase permeability, smooth-muscle-cell death may weaken the fibrous cap, and macrophage lipid handling influences inflammation and necrotic-core formation. Exercise simultaneously alters shear stress, lipoprotein metabolism, and immune tone, so lesion-level improvement cannot be assigned to one cell population from bulk tissue measurements. Lineage tracing and cell-specific perturbation will be needed to identify the exercise-responsive compartment.

### 4.5 Hypertension-related cardiovascular injury

Hypertension exposes the vasculature and myocardium to chronic mechanical load, neurohumoral activation, endothelial dysfunction, and oxidative stress. These factors can influence iron handling and lipid oxidation, but evidence for ferroptosis as a dominant mechanism is less developed than in ischemia-reperfusion or doxorubicin cardiotoxicity. Studies frequently report ROS, MDA, inflammatory mediators, and NRF2-related signaling; without broader ferroptosis assessment, these findings belong to Level C or D (Liu et al., 2023; Zhou et al., 2024). Current reviews therefore regard hypertensive ferroptosis as an emerging mechanism that requires broader marker panels and functional rescue experiments (Zhang and Guo, 2023; Liu et al., 2024).

Exercise training lowers blood pressure and improves vascular function through hemodynamic, autonomic, metabolic, and endothelial adaptations (Chen et al., 2022; Yang et al., 2022; Graham et al., 2024). Ferroptosis may contribute to injury in endothelial cells or the pressure-overloaded myocardium, but improvements in flow-mediated dilation, arterial stiffness, or heart-rate variability do not by themselves identify this form of regulated cell death (Zhang and Guo, 2023; Graham et al., 2024; Yang et al., 2025). Cardiovascular benefit and ferroptosis-specific evidence should therefore be reported as related but distinct outcomes.

## 5 Exercise modulation of ferroptosis-related cardiovascular biology

Because exercise studies of ferroptosis-related cardiovascular biology have been conducted in distinct injury models, this section evaluates the evidence according to disease context, mechanistic depth, and translational relevance. **Table 2** summarizes cardiovascular exercise studies that examined exercise interventions together with ferroptosis-related outcomes and classifies their evidential directness according to the framework described above.

### 5.1 Doxorubicin cardiotoxicity: most informative exercise-specific evidence

Doxorubicin cardiotoxicity provides the clearest exercise-specific test of ferroptosis-related cardioprotection because the injury involves mitochondrial iron stress, lipid-peroxide accumulation, and contractile dysfunction in relation to a defined toxic exposure (Tadokoro et al., 2020). In male mice, endurance exercise preconditioning reduced ferrous iron accumulation, lipid-peroxidation products, mitochondrial damage, and myocardial dysfunction. Importantly, this study incorporated ferrostatin-1, erastin, AMPKα2 manipulation, and cardiomyocyte experiments, providing the most informative exercise-specific causal evidence identified in this review (Wang L. et al., 2024).

Other aerobic-training studies during or after doxorubicin exposure also reported improved ventricular function, reduced myocardial injury, and coordinated changes in iron-handling, SLC7A11, GPX4, ACSL4, ferritin-related proteins, and lipid-peroxidation markers (Gao et al., 2024; Liu H. et al., 2025). These findings strengthen the consistency of the doxorubicin evidence base, although the degree of causal inference varies according to whether ferroptosis-sensitive rescue, induction, or core-pathway manipulation was included. Non-exercise studies of p62-dependent SLC7A11 degradation and NADPH oxidase inhibition further support the biological relevance of ferroptosis in this model (Wang J. et al., 2024; Wang et al., 2026).

### 5.2 Myocardial ischemia-reperfusion injury: timing-sensitive exercise evidence

Ischemia-reperfusion injury is a time-sensitive context in which exercise may influence ferroptosis-related susceptibility through antioxidant defense, mitochondrial preservation, iron handling, and inflammatory regulation. Because iron release, lipid-radical formation, mitochondrial permeability changes, and inflammatory amplification occur over different time scales, the timing of exercise exposure and tissue sampling is central to interpretation.

A 2025 mouse study identified IGFBP2 as an exercise-responsive circulating mediator after myocardial ischemia-reperfusion (Yang et al., 2025). Moderate treadmill training improved systolic function and exercise capacity and reduced infarct size and fibrosis. IGFBP2-null mice and cardiomyocyte IGF-1 receptor deletion weakened the benefit, while SIRT1 and TXNIP/TRX experiments mapped a downstream redox pathway. Cardiac and cellular assays included ferrous iron, lipid-peroxidation products, glutathione, GPX4, and viability. This design supports pathway involvement in the exercise response, although IGFBP2–SIRT1–TXNIP/TRX signaling also has broader metabolic and antioxidant functions.

Preconditioning, concurrent training, and post-infarction rehabilitation address different biological questions. Prior adaptation may raise resistance to a later insult, training during injury may modify ongoing stress responses, and rehabilitation after infarction mainly affects remodeling in surviving tissue. Serial sampling is therefore important for distinguishing an early ferroptosis-related burst from later inflammatory and fibrotic repair.

### 5.3 Diabetic cardiomyopathy and cardiac fibrosis: chronic remodeling contexts

Diabetic cardiomyopathy and fibrotic remodeling provide chronic disease contexts in which exercise may influence ferroptosis-related vulnerability through metabolic adaptation, mitochondrial quality control, redox regulation, and extracellular matrix remodeling. Compared with acute toxic or reperfusion injury, these models involve slower interactions among hyperglycemia, lipid metabolism, inflammation, oxidative stress, and cardiomyocyte or fibroblast responses.

In a high-fat-diet/streptozotocin model, cardiomyocyte NAT10 was required for the training-related improvement in systolic and diastolic function and for the maintenance of FTH1, SLC7A11, GPX4, and PTGS2 profiles (Shen et al., 2025). RNA immunoprecipitation sequencing linked NAT10-dependent N4-acetylcytidine modification to ferroptosis-related transcripts. Human explanted hearts supported the disease relevance of the pathway, although they did not test an exercise intervention.

In diabetic rats, moderate treadmill training improved cardiac pathology, mitochondrial morphology, lipid-peroxide indices, and function while engaging a proposed P4HA1–HIF-1α-NRF2/GPX4 pathway (Xie et al., 2026). Overexpression and knockdown experiments strengthened the pathway assignment, although direct measurements of prolyl-hydroxylase activity and HIF-1α turnover would help resolve the sequence. In vitamin D-deficient mice, 12 weeks of treadmill exercise activated cardiac VDR–NRF2–GPX4 signaling, reduced ferroptosis-associated changes, and limited fibrosis; cardiac-specific VDR knockdown weakened these effects (Cui et al., 2026). Together, these studies provide strong supportive evidence, while direct testing of core ferroptosis effectors would further strengthen causal interpretation.

### 5.4 HIIT and heart failure: emerging but not comparative evidence

HIIT is of interest because repeated high-intensity bouts impose strong metabolic and hemodynamic stimuli, followed by recovery periods that may promote mitochondrial adaptation, antioxidant remodeling, and vascular improvement. In male rats with isoprenaline-induced heart failure, eight weeks of HIIT used five four-minute bouts at 85%–90% of maximal oxygen uptake, separated by two minutes of active recovery at 50%–60% (Sabouri et al., 2025). Training improved ejection fraction, fractional shortening, fibrosis, and myocardial injury while shifting iron handling, SLC7A11, GPX4, NRF2, MDA, and endoplasmic-reticulum-stress measures in a favorable direction.

These findings support a ferroptosis-related interpretation when considered alongside functional cardiac improvement, but they do not establish that HIIT is superior to energy-matched moderate continuous training for ferroptosis-related cardiovascular outcomes. Whether HIIT produces distinct ferroptosis-related adaptations or reflects a different dose-intensity distribution remains unresolved.

### 5.5 Resistance and combined training: evidence gaps and biological plausibility

Resistance exercise and combined aerobic-resistance programs have established cardiovascular and metabolic value, including improvements in strength, blood pressure, body composition, endothelial function, and risk-factor control (Wewege et al., 2018; Alvarez et al., 2024). Their biological relevance to ferroptosis-related cardiovascular protection is plausible because they can influence skeletal-muscle metabolism, systemic inflammation, glucose handling, vascular function, myokine release, and redox adaptation. However, direct studies linking these modalities to tissue-level cardiovascular ferroptosis are still limited.

The current lack of direct cardiovascular ferroptosis studies does not exclude biological relevance, but it limits modality-specific conclusions. Circulating lipid-peroxidation outcomes from combined training remain general contextual evidence because they do not identify myocardial or vascular cell death (Ni et al., 2023). Future comparisons should distinguish local muscular adaptations, systemic metabolic responses, and myocardial or vascular ferroptosis-related changes rather than assume that systemic antioxidant improvement reflects cardiac ferroptosis modulation.

### 5.6 Indirect, non-cardiovascular, and human contextual evidence

Indirect evidence from neural, skeletal-muscle, metabolic, or general oxidative-stress studies can help identify conserved mechanisms, candidate mediators, and systemic exercise responses. These studies are useful for hypothesis generation, especially when they involve exerkines, mitochondrial quality control, iron transport, lipid peroxidation, or GPX4-independent defense pathways (Boström et al., 2012; Whitham et al., 2018; Jia et al., 2025). Treadmill training changed cortical iron handling and system Xc^−^–GPX4 signaling in APP/PS1 mice, while exercise preconditioning reduced neuronal ferroptosis through skeletal-muscle-derived exosomal miR-484 and ACSL4 regulation (Huang et al., 2024; Li C. et al., 2024). Aerobic training also enhanced NRF2-related defense in an aging model (Huang et al., 2024). Their relevance to myocardial or vascular ferroptosis remains inferential unless cardiovascular tissue, disease models, or pathway-specific endpoints are examined.

Human exercise studies provide translational context by showing improvements in endothelial function, cardiorespiratory fitness, blood pressure, metabolic control, inflammation, and oxidative-stress markers (Hambrecht et al., 1998; Wisløff et al., 2007; Gounder et al., 2012; Ellingsen et al., 2017; Wewege et al., 2018; Ni et al., 2023; Alvarez et al., 2024; Liu Z. et al., 2025; Zhang W. et al., 2025). These outcomes are clinically meaningful, but they are better interpreted as systemic contextual evidence rather than stand-alone evidence of ferroptosis inhibition in patients. This distinction is important because circulating oxidative-stress markers may reflect multiple tissues and processes. Because these studies do not directly demonstrate ferroptosis modulation in cardiovascular tissue, they are summarized separately in **Table 3** as indirect, general oxidative-stress, or human contextual evidence.

**Table 3:** Indirect, general oxidative-stress, and human contextual evidence.

Reference	Evidence type	Model/population and prescription	Main findings	Classification and interpretation
(Wewege et al., 2018)	Non-cardiovascular animal	Male APP/PS1 and wild-type mice, n=20/group; treadmill 12–15 m/min, 60 min/day, 5 days/week for 8 weeks	Altered cortical iron handling and increased Xc^−^/GPX4 with improved cognition	C. Direct neural-tissue study; cannot be treated as cardiac or vascular evidence.
(Alvarez et al., 2024)	Non-cardiovascular animal	Rat exercise preconditioning before cerebral ischemia; detailed exercise dose not reproduced here	Skeletal-muscle exosomal miR-484 regulated neuronal ACSL4 and ferroptosis-related injury	C. Supports inter-organ signaling but not cardiovascular ferroptosis.
(Huang et al., 2024)	Cardiac general oxidative-stress evidence	Young and aged male mice; moderate treadmill training (10 m/min, 50 min/day, 7% grade) for 6 weeks	Restored myocardial NRF2 and antioxidant enzymes in aged mice; intense endurance stress was less adaptive	D for ferroptosis; valuable dose-response context.
(Ni et al., 2023)	Human acute exercise	15 sedentary young adults (10 male/5 female); randomized crossover HIIT, moderate continuous exercise, and rest	Improved flow-mediated dilation, shear rate, lactate, VEGF, IGF-1, and irisin	D. Human vascular benefit without ferroptosis measurement.
(Zhou et al., 2024)	Human systematic review	Seven trials in older adults; exercise with or without antioxidant supplementation	Combined aerobic plus low-intensity resistance exercise ranked favorably for circulating lipid-peroxidation outcomes	D. Systemic lipid-peroxidation evidence; no cardiovascular tissue ferroptosis.
(Zhang X. et al., 2024)	Human systematic review/meta-analysis	Adults with metabolic syndrome; aerobic, resistance, or combined training >4 weeks	Aerobic exercise improved several cardiovascular risk factors; limited evidence for resistance/combined comparisons	Clinical context only; not ferroptosis evidence.
(Jia et al., 2025)	Human systematic review/meta-analysis	Eleven randomized trials and 904 participants with heart failure	HIIT reduced BNP/NT-proBNP compared with controls; inflammatory marker effects were not significant	Clinical feasibility and outcome context only; no ferroptosis measurement

Categories describe the directness of ferroptosis evidence rather than overall study quality. Human reviews and meta-analyses are included only to contextualize clinical exercise outcomes and were not treated as independent mechanistic evidence for ferroptosis. Non-cardiovascular animal studies were interpreted as indirect mechanistic context and were not considered direct evidence of cardiovascular ferroptosis.

APP/PS1, amyloid precursor protein/presenilin 1; BNP, B-type natriuretic peptide; GPX4, glutathione peroxidase 4; HIIT, high-intensity interval training; IGF-1, insulin-like growth factor 1; NRF2, nuclear factor erythroid 2-related factor 2; NR, not reported; NT-proBNP, N-terminal pro-B-type natriuretic peptide; VEGF, vascular endothelial growth factor.

### 5.7 Exercise dose, timing, and modality-specific interpretation

Exercise dose and timing are central to interpreting ferroptosis-related outcomes. Preconditioning before injury, exercise during toxic exposure, early rehabilitation after injury, and long-term training in chronic disease address different biological questions. Acute exercise may transiently increase redox turnover, whereas repeated training may improve resting mitochondrial resilience, antioxidant defense, vascular function, and metabolic flexibility.

The response may also be modified by baseline iron status, selenium availability, dietary fatty acids, age, sex, disease severity, chemotherapy, statins, renin-angiotensin-system blockers, sodium-glucose cotransporter 2 inhibitors, and antioxidant supplementation (Gomez-Cabrera et al., 2008; Ristow et al., 2009; Gounder et al., 2012; Muthusamy et al., 2012). Current evidence therefore supports protocol- and disease-specific interpretations rather than a general hierarchy of exercise modalities for ferroptosis-related cardiovascular biology. The most useful future comparisons will match total work, intensity distribution, recovery, and disease stage while measuring tissue-level ferroptosis-related pathways and cardiovascular function in parallel.

## 6 Discussion and translational priorities

### 6.1 Current state of the evidence

The cardiovascular exercise-ferroptosis literature has moved beyond isolated changes in malondialdehyde or GPX4, but its causal depth remains uneven. Endurance preconditioning in doxorubicin cardiotoxicity currently provides the most informative exercise-specific evidence because it links exercise exposure, ferroptosis-sensitive pharmacology, AMPKα2 manipulation, cardiomyocyte experiments, tissue markers, and cardiac function (Wang L. et al., 2024). Other models provide strong supportive evidence when coordinated marker panels and cardiovascular endpoints move in the same direction, but they vary in the degree to which ferroptosis is tested as a necessary mechanism (Yang et al., 2022; Cai et al., 2023).

Differences across models are biologically expected. Doxorubicin cardiotoxicity is a relatively tractable setting because mitochondrial iron stress and lipid-peroxide injury develop in relation to a defined toxic exposure. Ischemia-reperfusion injury involves a narrower and more dynamic window, in which early iron release and lipid-radical formation may occur before later inflammatory amplification and repair. Diabetic cardiomyopathy, pressure overload, fibrosis, and heart failure represent more chronic settings in which metabolic stress, mitochondrial remodeling, inflammation, and extracellular-matrix changes may overlap with ferroptosis-related vulnerability. These differences help explain why exercise-associated molecular patterns are not identical across cardiovascular models (Yang et al., 2022; Cai et al., 2023; Fefelova et al., 2023). This model dependence is consistent with recent reviews showing that ferroptosis regulation varies by disease trigger, cellular compartment, and metabolic state (Fang et al., 2023; Dixon and Olzmann, 2024; Liu et al., 2024).

A recurring pattern across the stronger experiments is improved control of the labile iron pool, lower phospholipid-peroxide burden, preservation of the SLC7A11–GSH–GPX4 defense axis, improved mitochondrial integrity, and better cardiac structure or function. This convergence is important because ferroptosis is not defined by a single marker. The most persuasive interpretation emerges when iron handling, lipid-peroxide injury, antioxidant defense, and cardiovascular function shift in the same biological direction (Cai et al., 2023; Fefelova et al., 2023). Recent methodological discussions caution that GPX4 abundance, ROS, MDA, or total iron cannot independently establish ferroptotic death (Zhang and Guo, 2023; Dixon and Olzmann, 2024).

Exercise also differs from pharmacological ferroptosis inhibitors. It does not target a single molecular node; rather, it produces coordinated changes in mitochondrial function, antioxidant capacity, substrate metabolism, vascular function, inflammatory tone, autonomic regulation, and iron handling. This pleiotropic nature is a strength for cardiovascular protection, but it also makes mechanistic attribution more difficult. A reduction in ferroptosis-related markers after exercise may reflect direct modulation of iron-dependent lipid peroxidation, improved upstream metabolic resilience, reduced inflammatory amplification, or broader restoration of cellular homeostasis. Exercise-related mitochondrial remodeling provides a plausible upstream explanation for this pleiotropy (Zhang H. et al., 2024).

Current evidence is therefore best interpreted in a model- and protocol-specific manner. Aerobic training, HIIT, resistance exercise, and combined programs may share some redox and mitochondrial adaptations, but they differ in hemodynamic load, local hypoxia, substrate demand, inflammatory response, and recovery kinetics. These differences make it premature to assign a fixed ferroptosis-related mechanism to each exercise modality (Mohammadkhani et al., 2023). A balanced interpretation is that appropriately prescribed exercise may reduce ferroptosis-related susceptibility in selected cardiovascular injury settings by strengthening the cellular systems that restrain iron-dependent lipid peroxidation (Cai et al., 2023; Fefelova et al., 2023; Mohammadkhani et al., 2023).

### 6.2 Methodological priorities for mechanistic studies

More complete exercise reporting would substantially improve comparability. Exercise prescriptions should specify modality, intensity, frequency, session duration, intervention duration, progression, recovery, adherence, timing of the final bout, and tissue-collection window. Animal or participant characteristics, disease stage, systemic iron status, medication, diet, randomization, blinding, and outcome-specific sample size are also important. FITT-VP terminology would make protocols easier to reproduce and compare (Hoffmann et al., 2014; Slade et al., 2016; Percie Du Sert et al., 2020).

Sampling time is not merely a technical detail but a determinant of biological interpretation. Early sampling may capture iron release, lipid-radical formation, and acute antioxidant consumption, whereas later sampling may reflect inflammation resolution, mitochondrial repair, fibrosis, or compensatory antioxidant adaptation. A single terminal measurement may therefore miss the peak of ferroptosis-related injury or obscure whether exercise delayed the process, reduced its magnitude, accelerated recovery, or altered baseline tissue resilience. Serial sampling across acute, subacute, and chronic windows would strengthen mechanistic interpretation (Yang et al., 2022). Recent work on cell-death crosstalk also supports serial, rather than single-time-point, assessment after reperfusion (Du et al., 2025).

A practical minimum assay set would include at least one iron-domain measure, one lipid-peroxidation or oxidized-phospholipid measure, one antioxidant-defense measure, and one prespecified cardiovascular functional endpoint. Stronger designs would add ferroptosis-sensitive rescue, induction, or core-gene manipulation to test whether ferroptosis-related changes contribute to the cardiovascular phenotype (Yang et al., 2022; Cai et al., 2023). Oxidized-phospholipid lipidomics, C11-BODIPY oxidation, GPX4 activity, GSH/GSSG, mitochondrial ultrastructure, and cell viability provide more information together than any single marker (Drummen et al., 2002). Direct functional assessment of native GPX4 or other suppressor activity can further strengthen pathway attribution beyond protein abundance alone (Nakamura et al., 2024).

Cell type also matters. Cardiomyocytes, endothelial cells, vascular smooth muscle cells, macrophages, and fibroblasts differ in iron handling, membrane lipid composition, antioxidant capacity, mitochondrial demand, and susceptibility to ferroptosis. Bulk myocardial or vascular tissue analysis can obscure these differences. Cell-specific perturbation, spatial transcriptomics, targeted lipid imaging, single-cell sequencing, or lineage-informed experiments may clarify which cardiovascular compartments are most responsive to exercise (Kuppe et al., 2022; Wünnemann et al., 2025). Modern cell-biological models similarly emphasize organelle- and cell-context-specific ferroptosis sensitivity (Fang et al., 2023; Dixon and Olzmann, 2024).

Future models should better reflect clinical heterogeneity. Many preclinical studies still rely on young male rodents, whereas cardiovascular disease often occurs with aging, diabetes, renal dysfunction, obesity, cancer treatment, iron deficiency, statin use, renin-angiotensin-system blockade, sodium-glucose cotransporter 2 inhibitors, or antioxidant supplementation. Including both sexes, reporting systemic iron status, and modeling relevant comorbidities would strengthen translational interpretation (Fefelova et al., 2023; Wünnemann et al., 2025).

### 6.3 Human translation and biomarker development

Human translation should initially focus on biomarker validation rather than direct therapeutic claims. Exercise trials in patients and at-risk populations have already shown improvements in cardiorespiratory fitness, endothelial function, blood pressure, glycemic control, lipid metabolism, inflammatory tone, and quality of life. These outcomes are clinically meaningful, but they do not directly identify ferroptosis-related changes in cardiovascular tissue. Candidate circulating markers need to be anchored to tissue-level ferroptosis signatures in paired experimental models or clinically available biospecimens before they are used as indicators of exercise-modulated ferroptosis in patients (Kalra et al., 2022; Kuppe et al., 2022; Mentz et al., 2023). Recent cardiovascular reviews consistently identify the lack of validated, specific clinical biomarkers as a central translational limitation (Fang et al., 2023; Zhang and Guo, 2023; Liu et al., 2024).

A staged strategy is most realistic. First, candidate markers such as oxidized phospholipid species, lipid-peroxidation products, iron-regulatory proteins, ferritin-related measures, glutathione-related indices, extracellular-vesicle cargo, exerkines, and inflammatory mediators should be linked to tissue-level cardiovascular injury (Kalra et al., 2022; Kuppe et al., 2022; Mentz et al., 2023). Second, observational cohorts can test whether these signatures track ventricular dysfunction, vascular injury, or remodeling independently of inflammation, renal function, diet, medication, and systemic iron status. Third, exercise trials can evaluate whether validated signatures change in a dose-, timing-, or modality-sensitive manner.

The distinction between acute and chronic exercise responses is essential. An acute bout may transiently increase oxidative turnover or circulating lipid-peroxidation products, whereas repeated training may improve resting antioxidant capacity, mitochondrial quality control, endothelial function, and metabolic flexibility. Immediate post-exercise sampling may reflect acute redox flux, while delayed resting-state sampling after a training period may better reflect adaptation.

Human studies would be most informative when circulating molecular panels are paired with functional and imaging outcomes, such as echocardiography, cardiac magnetic resonance, vascular ultrasound, arterial stiffness, flow-mediated dilation, cardiopulmonary exercise testing, or clinically available tissue specimens (Kuppe et al., 2022). At this stage, exercise is best viewed as a broad physiological intervention that may improve the biological environment in which ferroptosis-related injury occurs, alongside established cardiovascular treatment and rehabilitation practice.

### 6.4 Limitations and scope of the review

This review is a mechanism-centered narrative synthesis designed to integrate heterogeneous evidence across cardiovascular models, exercise interventions, ferroptosis-related pathways, and translational studies. It does not provide a formal meta-analysis, duplicate screening, exhaustive study enumeration, or a validated risk-of-bias score. The evidence categories used here clarify mechanistic directness and are not intended to replace formal quality assessment.

The main limitations arise from the evidence base itself (Yang et al., 2022; Cai et al., 2023; Fefelova et al., 2023; Mohammadkhani et al., 2023). Most studies are preclinical; exercise protocols are heterogeneous; ferroptosis assays are not standardized; and sex, age, comorbidity, systemic iron status, diet, and medication exposure are often incompletely reported. Human evidence remains largely contextual because most trials measure vascular function, systemic oxidative stress, inflammation, or metabolism rather than tissue-level cardiovascular ferroptosis.

Publication bias is also plausible because positive pathway studies are more likely to appear than null or conflicting results. In addition, terminology across the field is not fully standardized, and some reports use ferroptosis-related markers without distinguishing ferroptotic cell death from broader oxidative stress, mitochondrial injury, or inflammatory remodeling. These issues do not negate the biological relevance of the field, but they require cautious interpretation.

Taken together, the evidence supports a focused hypothesis: appropriately prescribed exercise may improve iron handling, lipid-peroxide control, antioxidant defense, mitochondrial resilience, and inflammatory balance in selected cardiovascular injury contexts (Cai et al., 2023; Fefelova et al., 2023; Mohammadkhani et al., 2023). Testing this hypothesis with more rigorous mechanistic and translational designs will determine whether ferroptosis-related biology can become a useful target for exercise-based cardiovascular protection.

## 7 Conclusions

Ferroptosis provides a useful framework for connecting iron availability, oxidizable membrane phospholipids, antioxidant failure, mitochondrial stress, and cardiovascular injury. Exercise can influence each component, but a change in NRF2, GPX4, MDA, or ferritin alone is not enough to establish ferroptotic cell death. The most persuasive experiments combine cardiovascular function with convergent iron and phospholipid measurements and a perturbation that changes ferroptotic susceptibility.

Current evidence remains preclinical and disease-specific. Endurance exercise currently has the clearest causal support in doxorubicin cardiotoxicity. Ischemia-reperfusion, diabetic cardiomyopathy, vitamin D-deficient cardiac fibrosis, and experimental heart failure add mechanistically informative but still model-dependent evidence involving IGFBP2, NAT10, P4HA1, VDR, NRF2, and SLC7A11–GSH–GPX4 signaling. Direct cardiovascular evidence for resistance and combined training is still scarce, and no human exercise study has demonstrated ferroptosis inhibition in cardiac or vascular tissue.

Progress now depends on reproducible exercise dosing, sex-balanced and comorbidity-relevant models, core-pathway rescue or genetic tests, oxidized-phospholipid measurements, serial sampling, and human biomarker validation. At present, exercise is best interpreted as a promising modifier of ferroptosis-related cardiovascular biology, whereas its status as a clinically established ferroptosis-targeted therapy remains to be demonstrated.
